# ﻿Taxonomic studies of the ground beetle subgenus *Falcinebria* Ledoux & Roux, 2005 (Coleoptera, Carabidae, *Nebria*) from the Japanese Alps (central Honshu), Shikoku, and Kyushu, Japan

**DOI:** 10.3897/zookeys.1254.157095

**Published:** 2025-10-01

**Authors:** Kôji Sasakawa

**Affiliations:** 1 Laboratory of Zoology, Department of Science Education, Faculty of Education, Chiba University, 1-33 Yayoi-cho, Inage-ku, Chiba 263-8522, Japan Chiba University Chiba Japan

**Keywords:** Endophallus, identification key, male genitalia, *Nebria reflexa*, new distribution record, new species, redescription, taxonomy

## Abstract

Nebria (Falcinebria) reflexa Bates and its relatives are among the most diverse groups of the genus in Japan, but their diversity has not yet been fully elucidated. In this study, specimens from three regions that had not been covered previously—the Japanese Alps (central Honshu), Shikoku, and Kyushu—were examined. Descriptions of *Nebria dracocephala***sp. nov.** (type locality: Honshu, Mount Ryûtô-san) and *Nebria elephanta***sp. nov.** (type locality: Shikoku, Mount Tsurugi-san), a redescription of *Nebria hikosana* Habu (type locality: Kyushu, Mount Hiko-san), and new distribution records for *N. niohozana* Bates (Honshu, Mount Teraji-yama), *N. furcata* Sasakawa (Honshu, Mount Kurai-yama), *Nebria dichotoma* Sasakawa (Honshu, Hiraya-tôge Pass), and *N. hikosana* (Kyushu, Mount Seburi-san) are presented. Species identification was based on the morphology of the male genital endophallus, a membranous inner sac everted from the aedeagus. For *N. dracocephala*, *N. elephanta*, and *N. hikosana*, the endophallus morphology exhibited several notable features—marked intraspecific variation in the size and shape of the surface structures, such as lobes in *N. dracocephala*, and significant morphological modifications of the gonopore protrusion in *N. elephanta* and *N. hikosana*. These findings, together with information on related species from previous studies, provide new insights into the processes of differentiation and morphological evolution of *N. reflexa* and related species. A checklist and updated key to all Japanese species of Nebria (Falcinebria) are provided.

## ﻿Introduction

**Figure 1.**
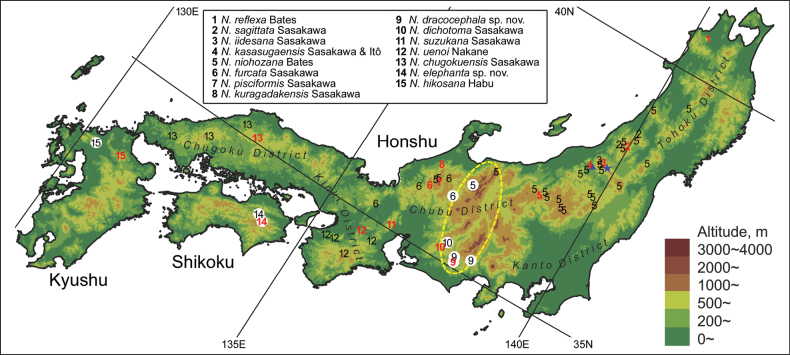
Distribution of species previously treated as *Nebria reflexa* on Honshu, Shikoku, and Kyushu, compiled from Sasakawa (2023b), Nakaya (2024), and new records (white circles). Only records with unambiguous species identity (i.e., collection sites of type materials and records based on specimens identified by the endophallus) are presented. Red letters denote the type localities of each species. The blue star indicates the locality where the sympatric occurrence of *N. iidesana* and *N. niohozana* was confirmed, and the blue triangle indicates the locality where the sympatric occurrence of *N. kasasugaensis* and *N. niohozana* was confirmed. The area enclosed by the yellow dashed ellipse indicates mountainous areas including the Japanese Alps.

**Figures 2–5.**
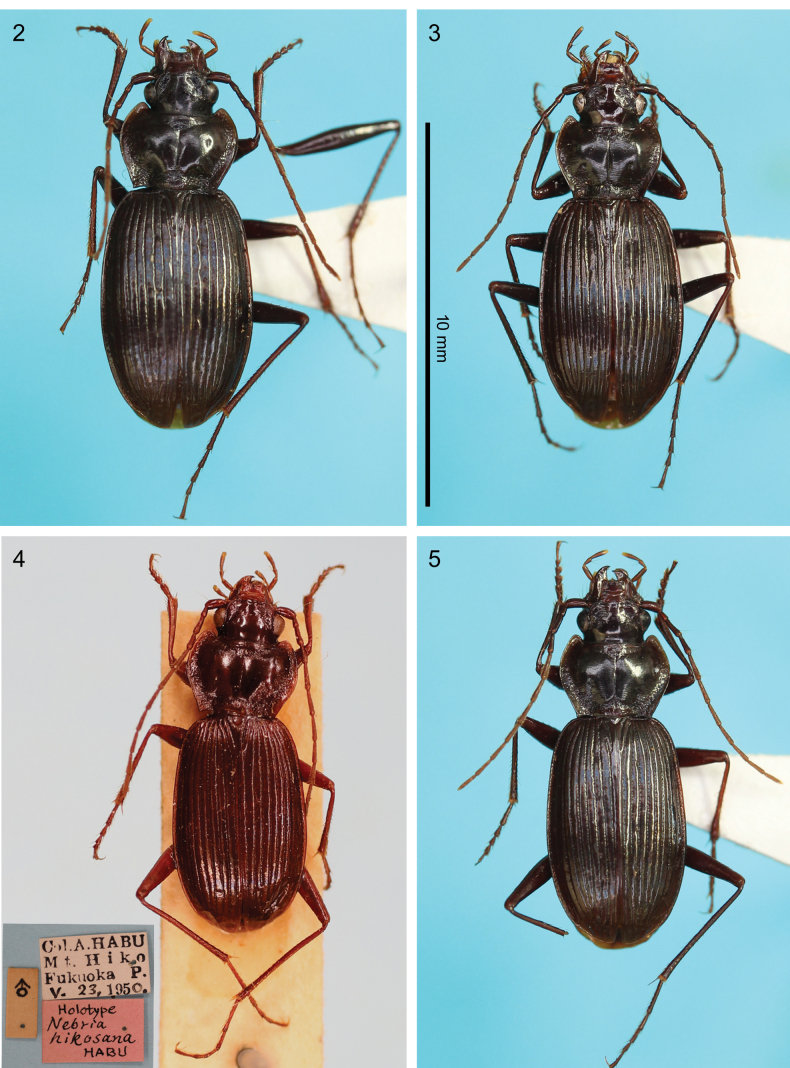
Habitus dorsal view of males of *Nebria* spp. 2. *N. dracocephala* sp. nov. holotype from Mount Ryûtô-san; 3. *N. elephanta* sp. nov. holotype from near Tsurugisan Ski Area; 4. *N. hikosana* holotype from Mount Hiko-san; 5. *N. hikosana* from Mount Seburi-san.

Falcinebria Ledoux & Roux, 2005 is a subgenus of the genus Nebria Latreille, 1802, endemic to East Asia, and is distributed in Japan, Taiwan, and mainland China (Huber 2017). This subgenus shows marked regional differentiation due to its poor dispersal ability, resulting from atrophied hind wings. In Japan, 14 species-group taxa are known from Honshu, Shikoku, and Kyushu (Fig. 1; Sasakawa 2023a, 2023b). Of the Japanese taxa, 11 had been treated as *Nebria reflexa* Bates, 1883 for several decades, and most were separated from the species and described as new species only recently (Sasakawa 2020, 2023b; Sasakawa and Itô 2021). Although these taxa are difficult to distinguish from each other by external morphology (Figs 2–5) and the easily observable superficial morphology of the genitalia (male aedeagus and parameres), they can be clearly distinguished by the endophallus of the male genitalia, which is a membranous inner sac everted from the aedeagus and has recently attracted attention for its taxonomic utility (Janovska et al. 2013). The distribution of these species is generally parapatric, and sympatric occurrences, consistent with reproductive isolation, have been confirmed in some species pairs at certain localities (Sasakawa 2020, 2023b; Sasakawa and Itô 2023). At such sympatric sites, differences in body size, which may reflect character displacement, are observed between the species (Sasakawa and Itô 2021). Thus, a series of recent studies have revealed hidden diversity in species previously treated as *N. reflexa*. This diversity suggests that these species could serve as a suitable model for elucidating the general mechanisms of differentiation and dispersal processes in insects with low dispersal ability. On the other hand, several issues remain, including areas where specimens have not been examined. In particular, for Kyushu, although a taxon was described previously based on specimens from a mountain in the northern part (Habu 1956), its taxonomic status has not yet been evaluated (Sasakawa 2020).

The present study on the taxonomy of this group was conducted to revise populations from three regions that had not been covered in previous studies (Fig. 1). The first region is the Japanese Alps, which consists mainly of the Hida, Kiso, and Akaishi mountains in central Honshu. Previous studies have examined only specimens from near the northern and southern ends of the Japanese Alps and have not examined specimens from most other parts of the mountain range (Sasakawa 2020). The second region is Shikoku. Given the remarkable geographic differentiation of this group in Honshu (Sasakawa 2023b) and the separation of Shikoku from Honshu and Kyushu by sea, the specificity of the Shikoku population is inferred. The third region is Kyushu. Although the taxon *hikosana* was described previously based on specimens from Mount Hiko-san (Habu 1956), no comparison with species from Honshu or examination of geographic differentiation within Kyushu has been conducted to date. A key to the Japanese species of the subgenus Falcinebria has also been updated based on the new taxonomic findings and is provided here. In addition, the processes of differentiation and morphological evolution of this group are discussed based on the present results and information on related species from previous studies.

## ﻿Materials and methods

Male specimens were identified by the morphology of the endophallus, which was everted and fully inflated by injecting toothpaste (White & White; LION, Tokyo, Japan) from the base of the aedeagus using an insulin syringe with a pre-attached 29-gauge needle (SS-10M2913; TERUMO, Tokyo, Japan). Females were identified by matching their external morphological features with those of identified males from the same collection site. Body length was measured from mandible apices to elytral end and is presented as a range (minimum–maximum) and mean ± standard deviation values for each species and sex. Terminology of endophallus structures followed Sasakawa (2020). Holotypes of the new species are deposited in the author’s collection deposited in the Laboratory of Zoology, Department of Science Education, Faculty of Education, Chiba University, Chiba, Japan (**KS**). Other specimens are deposited in the collections of Kitakyushu Museum of Natural History & Human History, Kitakyushu, Fukuoka, Japan (**KMNH**), the National Agriculture and Food Research Organization, Tsukuba, Ibaraki, Japan (**NARO**), and KS. For the endophallus of the *N. dichotoma* holotype male, scaled photographs taken in a previous study (Sasakawa 2020) were also used for comparisons. A list of the specimens examined and the Japanese names of some species reported here are provided in Suppl. material 1.

## ﻿Taxonomy

### ﻿ Nebria (Falcinebria) niohozana

Taxon classificationAnimaliaColeopteraCarabidae

Bates, 1883

31208C36-A96A-544D-ABF6-9C0C461E7FC3

Fig. 6

Nebria reflexa var. Niohozana : Bates (1883): 218 (original description; subgenus not specified; type locality: “Niohozan” (originally stated), changed to “Mikuni-toge Japan” through lectotype designation by Ledoux and Roux (1992)).
Nebria niohozana : Sasakawa (2020): 46 (redescription; subgenus Falcinebria).

#### Material examined.

3♂2♀ (KS), Japan • Mount Teraji-yama, Tobikoshi-shindô, on the border between Gifu Prefecture, Hida-shi, Kamioka-cho, Utsubo and Toyama Prefecture, Toyama-shi, Arimine, 4-X-2008, Hiroshi Nishida leg.

**Figures 6–9.**
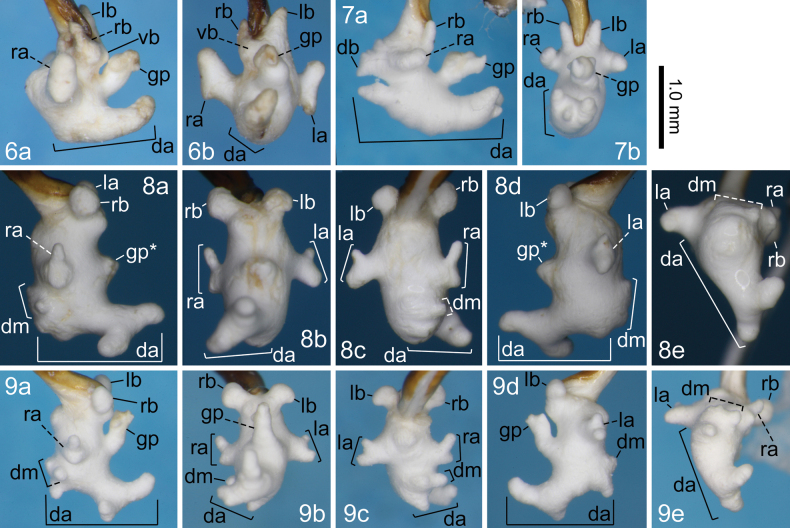
Right lateral (a), ventral (b), dorsal (c), left lateral (d), and posterodorsal (e) views of the endophallus of *Nebria* spp. 6. *N. niohozana* from Mount Teraji-yama; 7. *N. furcata* from Kuraiyama-tôge Pass; 8. *N. dichotoma* holotype from Mount Takanosu-yama; 9. *N. dichotoma* from near Hiraya-tôge Pass. Abbreviations: da, dorsoapical lobe; db, dorsobasal lobe; dm, dorsomedian lobe; gp, gonopore protrusion; la, left lateroapical lobe; lb, left laterobasal lobe; ra, right lateroapical lobe; rb, right laterobasal lobe; vb, ventrobasal swelling. Asterisk indicates that the gonopore protrusion or lobes are not fully everted.

#### Notes.

In the Japanese Alps, this species had been recorded only from Mount Shirouma-dake, near the northern end of the mountain range (Sasakawa 2020). The present collection record indicates that *N. niohozana* is more widely distributed in the northern part of the Japanese Alps than previously recognized. No distinct differences in the endophallus shape were found between the Shirouma-dake specimen and the present specimen.

### ﻿ Nebria (Falcinebria) furcata

Taxon classificationAnimaliaColeopteraCarabidae

Sasakawa, 2020

6CC93934-34BE-5F92-92A3-F95F93A6B42B

Fig. 7

Nebria furcata : Sasakawa (2020): 49 (original description; subgenus Falcinebria; type locality: “Arashiguchi, Kamiuchinami, Ôno-shi, Fukui Prefecture, Japan”).

#### Material examined.

1♂1♀ (KS), Japan • Gifu Prefecture, Gero-shi, Hagiwaracho, Yamanokuchi, Kuraiyama-tôge Pass, alt. 1100 m, 2-VII-2011, Kazue Ito leg.; 5♀ (KS), Japan • Gifu Prefecture, Gero-shi, Hagiwaracho, Yamanokuchi, Mount Kurai-yama, near the upper reaches of Araragi-ko Lake (Kuguno Disaster Prevention Dam), alt. 1100 m, 18-VI-2009, Hiroshi Nishida leg.

#### Notes.

This is the easternmost collection record of this species. The collection site is by definition not included in the Japanese Alps, but it is located in a mountain range adjacent to the northern part of the Japanese Alps (Fig. 1). The endophallus of the examined specimen was more similar to that of specimens from the distant type locality than to specimens from the nearest locality, Hida-shi (e.g., shapes of the dorsobasal lobe and the basal protrusion of the dorsoapical lobe), although the comparison was based on a small number of specimens from each locality (one or two males).

### ﻿ Nebria (Falcinebria) dichotoma

Taxon classificationAnimaliaColeopteraCarabidae

Sasakawa, 2020

EDA6550F-12D6-5ECF-9E06-4C07823B8EB5

Figs 8, 9

Nebria dichotoma : Sasakawa (2020): 52 (original description; subgenus Falcinebria; type locality: “Mount Takanosu (= Mount Dando), alt. 1000 m, Shitara-machi, Aichi Prefecture, Japan”).

#### Material examined.

1♂1♀ (KS), Japan • Aichi Prefecture, Toyota-shi, Otagicho, Dando-dani Valley, 27-V-1989, Hiroshi & Masami Nishida leg.; 2♂ (KS), Japan • Nagano Prefecture, Hiraya-mura, near Hiraya-tôge Pass, 14-VI-2002, Hiroshi & Takeyuki Nishida leg.

#### Notes.

Dando-dani Valley is located on the southern slope of Mount Takanosu-yama and can be regarded as virtually identical to the type locality. The record from Hiraya-tôge Pass is the northernmost collection record for this species, the first record outside the type locality, and the first record from Nagano Prefecture. The endophallus of the Hiraya-tôge Pass specimen differed slightly from that of specimens from the type locality in the shape of the backward sub-lobe at the apical 2/5 of the dorsoapical lobe (slightly bifid in the Hiraya-tôge Pass specimen versus simply rounded in the specimens from the type locality) and in the sizes of sub-lobes of the dorsomedian lobe and the base of the dorsoapical lobe. In the original description of *N. dichotoma*, the morphology of the gonopore protrusion could not be examined because of insufficient inversion of this structure. Therefore, the present result provides the first description of the gonopore protrusion of this species. Its gonopore protrusion was simple cylindrical, relatively long, and strongly bent at the base toward the base of the endophallus.

### ﻿ Nebria (Falcinebria) dracocephala sp. nov.

Taxon classificationAnimaliaColeopteraCarabidae

E757FAD8-A54A-5A04-BDDB-E8108042BCFC

https://zoobank.org/D7BA5BC8-B0F1-435E-886F-9B95BF08CAA6

Figs 2, 10–12

Nebria reflexa : Uéno (1985): 56 (part; subgenus not specified).

#### Type material.

***Holotype***: ♂ (KS), Japan • Shizuoka Prefecture, Hamamatsu-shi, Mount Ryûtô-san, alt. 1200 m, 3-X-2006 [no collector data]. ***Paratypes***: 1♀ (KS), same data as the holotype; 1♂ (KS), Japan • Shizuoka Prefecture, Shizuoka-shi, Aoi-ku, Mount Sasa-yama, alt. 1500 m, 29-X-2005, Masato Mori leg.; 2♂1♀ (KS), Japan • Shizuoka Prefecture, Tenryu-shi, Sakumacho, Mount Idoguchi-yama, the upper reaches of Aizuki-gawa River, 18-V-1996, Hiroshi Nishida leg.

**Figures 10–12.**
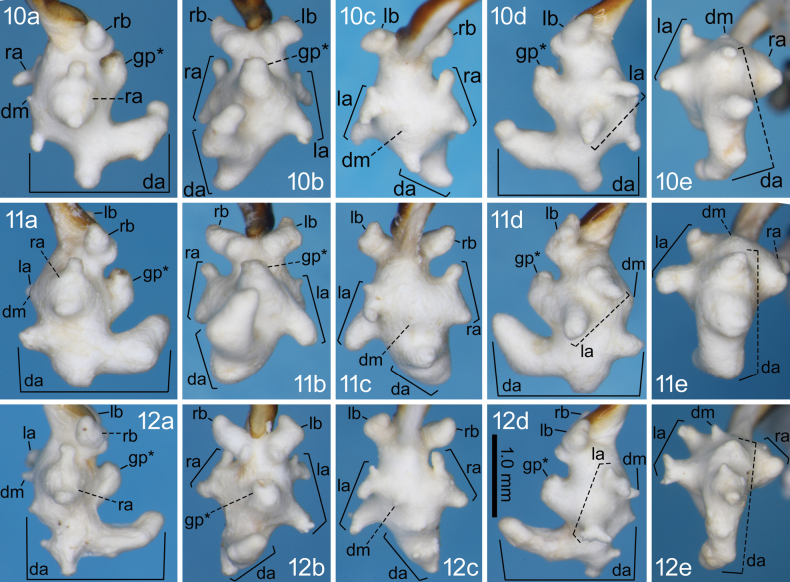
Right lateral (a), ventral (b), dorsal (c), left lateral (d), and posterodorsal (e) views of the endophallus of *Nebria dracocephala* sp. nov. from Mount Ryûtô-san (10), Mount Idoguchi-yama (11), and Mount Sasa-yama (12).

#### Diagnosis.

Similar to the locally adjacent species *N. dichotoma*, but distinguished by the shape of the endophallus, particularly the left lateroapical lobe composed of two sub-lobes and the dorsoapical lobe with apex not bifurcated (bifurcated in *N. dichotoma*).

#### Description.

Body length: ♂, 9.95–10.73 mm, 10.26 ± 0.34 mm (*n* = 4); ♀, 10.89–11.13 mm, 11.01 ± 0.17 mm (*n* = 2). Sternum 7 with two setae on each ventrolateral side in both male and female. Other external structures, coloration, and chaetotaxy as in other related species that had been previously regarded as *N. reflexa* (Sasakawa 2020). Dorsobasal lobe absent. Dorsomedian lobe undeveloped, with a minute, slender protrusion or a weak, wide swelling. Dorsoapical lobe with a simple-shaped (i.e., not bifurcated) protrusion at the basal part, the size of which varies among localities, ranging from smaller than that of the dorsobasal lobe to almost the same size as the laterobasal lobes; the dorsomedian area with a large protrusion, the apex of which is simply rounded or bifid, varying among localities; the apical portion bent ventrally, with the dorsal bend discontinuous in lateral view, more or less swollen. Laterobasal lobes large; the left lobe more rectangular in shape than the right in ventral view; at the distal end of both right and left lobes, the endophallus-base side corner more protruding than the opposite side; ventrobasal surface largely swollen on both right and left lobes, distinctly visible in lateral view. Ventrobasal swelling absent. Right lateroapical lobe markedly wide in dorsal view, with the endophallus-base and -apex side corners protruding, resulting in a T-shape in dorsal view; the endophallus-base side corner more slender and protruding than the opposite side. Left lateroapical lobe composed of two sub-lobes, one on the left dorsolateral side and the other on the left lateral side of the endophallus; the sub-lobe on the dorsolateral side T-shaped, with the endophallus-apex side corner more robust and protruding than the opposite side; the sub-lobe on the lateral side large, with a simply rounded or slightly bifid apex, varying among localities. Gonopore protrusion bent at the base, directed toward the endophallus base; the observable part simply curved cylindrical, with no additional structures such as protrusions. Relative sizes of some lobes and protrusions are as follows: dorsomedian lobe < endophallus-apex side corner of left dorsolateral sub-lobe of left lateroapical lobe ≈ endophallus-base side corner of right lateroapical lobe < swelling of ventrobasal surface of laterobasal lobes < left lateral sub-lobe of left lateroapical lobe ≤ laterobasal lobe (including swelling of ventrobasal surface) ≤ protrusion on dorsomedian area of dorsoapical lobe.

#### Notes.

There was marked variation in endophallus morphology, both qualitatively (shape) and quantitatively (size). However, all specimens shared basic structures, such as the left preapical lobe composed of two sub-lobes and the dorsoapical lobe with protrusions on the dorsobasal, dorsomedian, and dorsoapical sides, none of which are possessed by other closely related species (Sasakawa 2020, 2023b; Sasakawa and Itô 2021). Given their morphological complexity, these shared characters are probably synapomorphies uniting the three populations rather than symplesiomorphies. Therefore, the three populations were treated as a single species. Morita and Hirai (2010) recorded “*N. reflexa*” from the Abe-tôge Pass, 9 km northwest of Mount Sasa-yama, and this specimen is also most likely *N. dracocephala*.

#### Etymology.

The specific name is a combination of the Latin *draco* (dragon) and the Latin *cephalus*, -*a*, -*um* (head), derived from the type locality Ryûtô-san, which means “dragon’s head mountain” in Japanese.

### ﻿ Nebria (Falcinebria) elephanta sp. nov.

Taxon classificationAnimaliaColeopteraCarabidae

6A53FAF5-680D-5956-AB01-86EB6E00DCDF

https://zoobank.org/3F7263F6-EFD2-4190-B3FF-5664891B62A6

Figs 3, 13

Nebria reflexa : Uéno (1985): 56 (part; subgenus not specified).

#### Type materials.

***Holotype***: ♂ (KS), Japan • Tokushima Prefecture, Tsurugi-cho, Ichiu, near Tsurugisan Ski Area, alt. 1000 m, 14-VII-2006, Masato Mori leg. ***Paratypes***: 1♂1♀ (KS), Japan • Tokushima Prefecture, Higashimiyoshi-cho, Nishisho, near Sajiki-tôge Pass, alt. 800 m, 10-VII-1999, Masato Mori leg.

**Figures 13–15.**
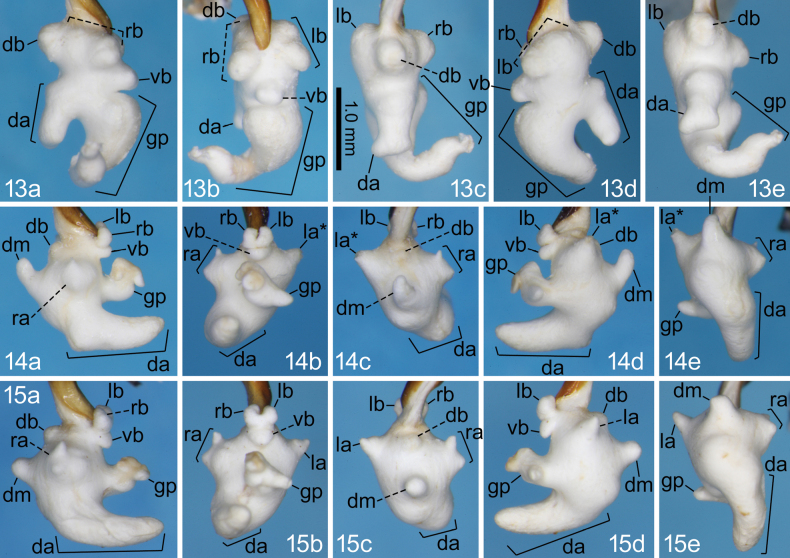
Right lateral (a), ventral (b), dorsal (c), left lateral (d), and posterodorsal (e) views of the endophallus of *Nebria* spp.; 13. *N. elephanta* sp. nov. holotype from near Tsurugisan Ski Area; 14. *N. hikosana* from Ryûmon-kyô Gorge; 15. *N. hikosana* from Mount Seburi-san.

#### Diagnosis.

Similar to the locally adjacent species *N. chugokuensis* Sasakawa, 2020 and *N. uenoi* Nakane, 1963, but distinguished from *N. chugokuensis* by a smaller body size (in *N. chugokuensis*, 9.97–10.32 mm for males and 10.69–11.75 mm for females; Sasakawa 2020) and the absence of a concavity on the ventral surface of the aedeagal apex (present in *N. chugokuensis*), and from *N. uenoi* by the shape of the endophallus, in particular, the developed gonopore protrusion (undeveloped in *N. uenoi*) and the absence of lateroapical lobes on both sides (present in *N. uenoi*).

#### Description.

Body length: ♂, 9.70–9.81 mm, 9.75 ± 0.08 mm (*n* = 2); ♀, 10.54 mm (*n* = 1). Sternum 7 with two setae on each ventrolateral side in the male, and three in the female. Other external structures, coloration, and chaetotaxy as in other related species that had been previously regarded as *N. reflexa* (Sasakawa 2020). Dorsobasal lobe large, semi-spherical. Dorsomedian lobe absent. Dorsoapical lobe scalene trapezium-shaped in dorsal view, with the left and right sides concave; the left side more concave than the right; the left apical corner more protruding than the right. Laterobasal lobes with the ventrobasal surface largely swollen in a semi-spherical shape; the other part less swollen but wide, reaching the base of the dorsobasal lobe. Ventrobasal swelling large, semi-ellipsoid. Lateroapical lobes absent on both right and left sides. Gonopore protrusion markedly large and long, with total length along the median line from the base of the protrusion to the gonopore (apical end of the protrusion) longer than that from the base of the endophallus (ostium of the aedeagus) to the base of the protrusion; basal half thick, oriented in the same direction as the median line of the endophallus; apical half slender, strongly bent right laterally. Relative sizes of some lobes and protrusions are as follows: ventrobasal swelling ≤ swelling of ventrobasal surface of laterobasal lobes ≈ dorsobasal lobe < dorsoapical lobe < basal half of gonopore protrusion.

#### Notes.

The description of setae on female sternum 7 as three pairs was based on only one specimen. This character state must be confirmed based on additional specimens, because in Carabidae, the number of setae on the body surface, including sternum 7, often varies due to additional setae, etc. The present collection sites are located on the northern slope of Mount Tsurugi-san in the eastern part of Shikoku. Considering the relatively wide distribution of related species and the continuity of the forest environment, which seems to be suitable for this group, *N. elephanta* is assumed to be widely distributed around Mount Tsurugi-san. In Shikoku, there is also a record of “*N. reflexa reflexa*” from Mount Takanawa-san in the western part, 110 km from Mount Tsurugi-san (Yoshitomi et al. 2012). The identity of the Takanawa-san specimens remains to be clarified in a future study.

#### Etymology.

The specific name is derived from the Latin *elephantus*, -*a*, -*um* (elephant) and refers to the elongated gonopore protrusion of this species, which resembles the trunk of an elephant.

### ﻿ Nebria (Falcinebria) hikosana

Taxon classificationAnimaliaColeopteraCarabidae

Habu, 1956

E658C369-4B15-5A09-8FF8-0810DB7AEE98

Figs 4, 5, 14, 15

Nebria hikosana : Habu (1956): 170 (original description; subgenus not specified; type locality: “Mt. Hiko, Fukuoka Prefecture, Japan”); Farkač and Janata (2003): 94 (subgenus Orientonebria).
Nebria reflexa hikosana : Nakane (1963): 19 (subgenus Paranebria); Uéno (1985): 57 (subgenus not specified); Ledoux and Roux (2005): 830 (subgenus Falcinebria); Nishida (2005): 2 (subgenus not specified); Yoshitake et al. (2011): 32 (subgenus Falcinebria); Huber (2017): 50 (subgenus Falcinebria).

#### Materials examined.

• ***Holotype*** ♂ (NARO), “Col.A.HABU / Mt. Hiko / Fukuoka P. / V. 23, 1950 // Holotype / Nebria / hikosana / HABU // ♂”

#### Additional material.

3♂ (KMNH), Japan • Fukuoka Prefecture, Soeda-machi, Hikosan, Mount Hiko-san, Yasuo Takakura leg. (1♂ [no further locality information], 8-VI-1982; 2♂, Ryûmon-kyô Gorge, 8-V-1989); 4♂5♀ (KS), Japan • Saga Prefecture, Kanzaki-shi, Mount Seburi-san, alt. 950 m, 15-V-2001, Masato Mori leg.; 1♀ (KMNH), Japan • Fukuoka Prefecture, Fukuoka-shi~Saga Prefecture, Kanzaki-shi, Mount Seburi-san, 7-V-1977, Munemichi Fukamachi leg.

#### Diagnosis.

Similar to the locally adjacent species *N. chugokuensis*, but distinguished by one pair of setae on the ventral side of the sterna 7 in the male (two pairs in *N. chugokuensis*) and the absence of a concavity on the ventral surface of the aedeagal apex (present in *N. chugokuensis*).

#### Redescription.

Body length: ♂, 9.14–9.92 mm, 9.64 ± 0.27 mm (*n* = 8); ♀, 10.27–10.89 mm, 10.56 ± 0.24 mm (*n* = 6). Sternum 7 with one seta on each ventrolateral side in the male, and two in the female. Other external structures, coloration, and chaetotaxy as in other related species that had been previously regarded as *N. reflexa* (Sasakawa 2020). Dorsobasal lobe small and slightly swollen. Dorsomedian lobe semi-prolate-spheroid, weakly bent toward the endophallus base or not bent and simply directed dorsally. Dorsoapical lobe elongated, apically narrowed gradually, weakly and uniformly bent ventrally; apex widely rounded; dorsobasal surface slightly swollen in some specimens. Laterobasal lobes small, nearly spherical; in ventral view, the left and right laterobasal lobes nearly attached to each other, concealing the aedeagus apex. Ventrobasal swelling small, semi-spheroid, attached to the fused basal portion of the left and right laterobasal lobes in ventral view. Right lateroapical lobe moderate in size, bifurcated in a V-shape in dorsal view; subapical lobes both conical and about the same size. Left lateroapical lobe conical, except for the surface on the endophallus-base side in dorsal/ventral view, which is slightly swollen. Gonopore protrusion large, directed ventrally, with an additional protrusion on the left side at the middle; additional protrusion straight, directed left laterally, gradually narrowed apically, with a length more than half that of the main body of the gonopore protrusion. Relative sizes of some lobes and protrusions are as follows: subapical lobes of the right lateroapical lobe < laterobasal lobes ≈ ventrobasal swelling < additional protrusion of the gonopore protrusion ≤ dorsomedian lobe < main body of the gonopore protrusion < dorsoapical lobe.

#### Notes.

There is disagreement as to whether the taxon *hikosana* should be treated as a subspecies of *N. reflexa* or as a distinct species, but neither treatment has been based on concrete evidence. Here, *hikosana* is regarded as a distinct species rather than a subspecies of another species, based on marked differences in endophallus morphology compared with other related species, particularly *N. chugokuensis*, the closest distributed species to *N. hikosana* (Sasakawa 2020). In Kyushu, there is also a record of this species from Mount Shaka-dake, located 30 km south of Mount Hiko-san and belonging to a different mountain range (Imasaka et al. 2019), but its identity requires confirmation in future studies.

##### ﻿Checklist of Japanese Nebria (Falcinebria) and distribution

*N. chugokuensis* Sasakawa, 2020; Honshu, Chûgoku Mountains

*N. dichotoma* Sasakawa, 2020; Honshu, Mount Takanosu-yama and adjacent mountainous area

*N. dracocephala* sp. nov.; Honshu, Mounts Ryûtô-san, Idoguchi-yama, and Sasa-yama

*N. elephanta* sp. nov.; Shikoku, Mount Tsurugi-san

*N. furcata* Sasakawa, 2020; Honshu, mountainous areas around Hakusan and Hira Mountains, ranging from Mount Teraji-yama in the east to Mount Uchimi-yama in the west

*N. hikosana* Habu, 1956; Kyushu, Mounts Hiko-san and Seburi-san

*N. iidesana* Sasakawa, 2020; Honshu, Iide Mountains

*N. kasasugaensis* Sasakawa & Itô, 2021; Honshu, Mount Kasasuga-yama and adjacent mountainous area

*N. kobushicola* Sasakawa, 2023; Honshu, Yatsugatake and Okuchichibu mountains

*N. kuragadakensis* Sasakawa, 2020; Honshu, Mount Kuraga-dake and adjacent mountainous area

*N. niohozana* Bates, 1883; Honshu, mountainous areas mainly on and near the Sea of Japan side, ranging from Mount Mahiru-dake in the north to the Hakusan Mountains in the west

*N. pisciformis* Sasakawa, 2020; Honshu, Ôshirakawa-dani, a valley east of the Hakusan Mountains

*N. reflexa* Bates, 1883; Honshu, Mount Iwaki-san

*N. sagittata* Sasakawa, 2020; Honshu, Asahi Mountains and Mount Gassan.

*N. suzukana* Sasakawa, 2023; Honshu, Suzuka Mountains

*N. taketoi* Habu, 1962; Honshu, Northern Hida Mountains

*N. uenoi* Nakane, 1963; Honshu, Mountainous areas of the Kii Peninsula

### ﻿Key to species of Nebria (Falcinebria) from Japan (for males only)

This key is an updated version of that in Sasakawa (2023b), with the addition of *N. dracocephala*, *N. elephanta*, and *N. hikosana*. In the subgenus Falcinebria, it is virtually impossible to identify species based solely on female specimens. Usually, females are identified based on conspecific males from the same collection site. Therefore, the key is provided for males only.

**Table:** 

1	Pronotum strongly cordate; hind angles acute (Sasakawa 2023a: fig. 3E–H). Elytra widest almost at the middle, with anterior and lateral margins smoothly connected, forming an arc (Sasakawa 2023a: fig. 3A–D). Sterna 4–6 with 2–6 setae on each ventrolateral side	**2**
–	Pronotum less cordate; hind angles square to somewhat acute (Figs 2–5; Sasakawa 2020: figs 5–16). Elytra widest slightly behind the middle, with anterior and lateral margins less smoothly connected (Figs 2–5; Sasakawa 2020: figs 5–16). Sterna 4–6 with 1–3 (usually 2) setae on each ventrolateral side	**3**
2	Pronotum anterior angles more produced (Sasakawa 2023a: fig. 3G ,H). Laterobasal lobes of endophallus semi-spherical, directed ventrally (Sasakawa 2023a: fig. 5, lb and rb). Lateroapical lobes T-shaped in dorsal view (Sasakawa 2023a: fig. 5, la and ra)	***N. kobushicola***
–	Pronotum anterior angles less produced (Sasakawa 2023a: fig. 3E, F). Laterobasal lobes of endophallus semi-ellipsoid, directed ventrobasally (Sasakawa 2023a: fig. 4, lb and rb). Lateroapical lobes broadly rounded at apex, directed laterally (Sasakawa 2023a: fig. 4, la and ra)	***N. taketoi***
3	Male sternum 7 with one seta on each ventrolateral side. Gonopore protrusion of endophallus with an additional protrusion on the left side in the middle; the additional protrusion straight, directed left laterally, gradually narrowed apically (Figs 14, 15, gp). Laterobasal lobes small, nearly spherical (Figs 14, 15, lb and rb). Ventral swelling small, semi-spheroid (Figs 14, 15, vb). Right lateroapical lobe V-shaped in dorsal view (Figs 14, 15, ra). Left lateroapical lobe almost conical (Figs 14, 15, la)	***N. hikosana***
–	Male sternum 7 with two setae on each ventrolateral side. Gonopore protrusion of endophallus without additional protrusion (e.g., Figs 6–12, gp)	**4**
4	Gonopore protrusion of endophallus larger than any other lobe/protrusion on the endophallus surface; basal half thick; apical half slender, strongly bent right laterally; total length along the median line longer than the distance from endophallus base to gonopore protrusion base (Fig. 13, gp). Both right and left lateroapical lobes absent	***N. elephanta* sp. nov.**
–	Gonopore protrusion of endophallus not enlarged; size about the same as or smaller than other lobes/protrusions on the endophallus surface (e.g., Figs 6–12, gp). Both right and left lateroapical lobes present (Figs 6–12, la and ra)	**5**
5	Ventral surface of aedeagal apex deeply concave (Sasakawa 2020: fig. 35, ac). Lateroapical lobes of endophallus bifurcated at the base, with the larger apex further bifurcated (Sasakawa 2020: fig. 35, la and ra). Dorsoapical lobe large, arc-shaped in lateral view, with posterior and anterior ends simple (Sasakawa 2020: fig. 35, da). Ventrobasal swelling large, semi-spherical in lateral view (Sasakawa 2020: fig. 35, vb)	***N. chugokuensis***
–	Ventral surface of aedeagal apex not deeply concave (e.g., Figs 6–15)	**6**
6	Dorsobasal lobe of endophallus present (e.g., Fig. 7a, db)	**7**
–	Dorsobasal lobe of endophallus absent (e.g., Fig. 8a)	**11**
7	Left lateroapical lobe of endophallus bifurcated at the base (Sasakawa 2023c: figs 2–5, la). Right lateroapical lobe cylindrical, not bifurcated (Sasakawa 2023c: figs 2–5, ra). Laterobasal lobes larger than the right lateroapical lobe (Sasakawa 2023c: figs 2–5, lb and rb). Dorsomedian lobe present (Sasakawa 2023c: figs 2–5, dm)	***N. uenoi***
–	Neither right nor left lateroapical lobe of endophallus bifurcated (e.g., Fig. 7, la and ra). Dorsomedian lobe absent	**8**
8	Dorsobasal lobe of endophallus smaller than laterobasal lobes (Sasakawa 2023b: fig. 3A–F, db). Right lateroapical lobe semi-spherical, larger than dorsobasal lobe (Sasakawa 2023b: fig. 3A–F, ra)	***N. suzukana***
–	Dorsobasal lobe of endophallus larger than laterobasal lobes (e.g., Fig. 7, db; Sasakawa 2020: figs 27–30, db). Right lateroapical lobe bent ventrally, smaller than dorsobasal lobe (e.g., Fig. 7, ra; Sasakawa 2020: figs 27–30, ra)	**9**
9	Left lateroapical lobe of endophallus not bifurcated (Fig. 7, la). Dorsoapical lobe with apical margin divided into three projections in dorsal view of apical part (Fig. 7, da)	***N. furcata***
–	Left lateroapical lobe of endophallus bifurcated at the base (Sasakawa 2020: figs 29, 30, la). Apical margin of dorsoapical lobe not divided or only ambiguously divided (Sasakawa 2020: figs 29, 30, da)	**10**
10	Left lateroapical lobe of endophallus with sub-lobe on the apical side larger than the sub-lobe on the basal side (Sasakawa 2020: fig. 30, la)	***N. kuragadakensis***
–	Left lateroapical lobe of endophallus with sub-lobe on the apical side smaller than the sub-lobe on the basal side (Sasakawa 2020: fig. 29, la)	***N. pisciformis***
11	Neither right nor left lateroapical lobe of endophallus bifurcated (e.g., Sasakawa and Itô 2021: figs 2–4, la and ra)	**12**
–	Right and/or left lateroapical lobes of endophallus bifurcated (e.g., Figs 6, 8–12, la and ra)	**14**
12	Dorsoapical lobe of endophallus with apex simple, not bifurcated (Sasakawa and Itô 2021: fig. 2, da). Right lateroapical lobe directed laterally, with apex widely rounded (Sasakawa and Itô 2021: fig. 2, ra)	***N. kasasugaensis***
–	Dorsoapical lobe of endophallus with apex bifurcated (Sasakawa and Itô 2021: figs 3, 4, da)	**13**
13	Right lateroapical lobe of endophallus with apex bent anteriorly (Sasakawa 2020: figs 18, 19, ra). Basal protrusion of the dorsoapical lobe with length longer than twice the width at the base; the left apex of the dorsoapical lobe more than twice the size of the right apex in dorsal view (Sasakawa 2020: figs 18, 19, da)	***N. sagittata***
–	Right lateroapical lobe of endophallus with apex wide, not bent in dorsal view (Sasakawa 2020: figs 20, 21, ra). Basal protrusion of the dorsoapical lobe with length shorter than 1.5 times the width at the base; right and left apices of the dorsoapical lobe almost similar in size (Sasakawa 2020: figs 20, 21, da)	***N. iidesana***
14	Right lateroapical lobe of endophallus bifurcated in a T- or Y-shape in dorsal view (e.g., Fig. 6, ra). Body length > 9.4 mm (e.g., Fig. 2)	**15**
–	Right lateroapical lobe of endophallus not bifurcated; semi-spherical, except for an apical protrusion, which is bent in the basal direction (Sasakawa 2020: fig. 17, ra). Left lateroapical lobe bifurcated at the base (Sasakawa 2020: fig. 17, la). Laterobasal lobes smaller than the right lateroapical lobe (Sasakawa 2020: fig. 17, la and ra). Dorsomedian lobe present (Sasakawa 2020: fig. 17, da). Body length < 9.1 mm	***N. reflexa***
15	Left lateroapical lobe of endophallus composed of two sub-lobes, one on the left dorsolateral side and the other on the left lateral side of the endophallus (Figs 10–12, la). Dorsomedian lobe markedly small, not bifurcated (Figs 10–12, da)	***N. dracocephala* sp. nov.**
–	Left lateroapical lobe of endophallus T- or Y-shaped, almost symmetrical with the right lateroapical lobe in dorsal view (e.g., Figs 6, 8, 9, la and ra)	**16**
16	Dorsoapical lobe of endophallus with apex bifurcated in a Y-shape (Figs 8, 9, da). Laterobasal lobes spherical, larger than lateroapical lobes (Figs 8, 9, la, lb, ra, rb)	***N. dichotoma***
–	Dorsoapical lobe of endophallus with apex not bifurcated (e.g., Fig. 6, da). Laterobasal lobes cylindrical, smaller than lateroapical lobes (e.g., Fig. 6, la, lb, ra, rb)	***N. niohozana***

## ﻿Discussion

This study described new species from the southern Japanese Alps and Shikoku, and provided a redefinition and a new distribution record outside the type locality of *N. hikosana*. New distribution records were also reported for three known species from the Japanese Alps and surrounding areas, with some of these expanding the known distributions of the species. These results were all based on comparative morphology of the endophallus, thereby reaffirming the taxonomic utility of the morphology of this male genital structure.

The observed endophallus structures, together with information obtained from previous studies, provide new insights not only into the taxonomy of each species, but also into the processes of differentiation and morphological evolution of *N. reflexa* and related species. *Nebria dracocephala* shows notable intraspecific variation in endophallus morphology. The endophallus structures, such as lobes, swellings, and protrusions, exhibited marked individual variations not only in size (quantitative component) but also in shape (qualitative component) in this new species. For example, the protrusion at the dorsomedian area of the dorsoapical lobe was bifurcated apically in the Sasa-yama specimen but simply rounded without bifurcation in the Ryûtô-san and Idoguchi-yama specimens. Furthermore, the lateral sub-lobe of the left lateroapical lobe was bifid in the Sasa-yama specimen but not in the Ryûtô-san and Idoguchi-yama specimens. Interindividual variation was also observed between the Ryûtô-san and Idoguchi-yama specimens; for example, considerable size variations in the protrusions and swellings on the dorsal side of the dorsoapical lobe, and the presence or absence of the dorsomedian lobe. Similar marked intraspecific variations in the endophallus shape were also reported in *N. uenoi*. Similar to *N. dracocephala*, there are considerable variations in the shape and size of some protrusions and swellings on the dorsal side of the endophallus in *N. uenoi*, and some variations were reported to exhibit geographically restricted distribution patterns (Sasakawa 2023c). Importantly, such remarkable variation in endophallus morphology observed in *N. dracocephala* and *N. uenoi* is exceptional among related species. For example, in *N. niohozana*, *N. furcata*, and *N. chugokuensis*, photographs of the endophallus from several populations were provided in this and previous studies (Hayashi 2020; Sasakawa 2020, 2023b; Sasakawa and Itô 2021; Nakaya 2024). Examination of these photographs shows no clear intraspecific variation in the endophallus shape in these three species, although all have a wider distribution than *N. dracocephala* and *N. uenoi*. Both *N. dracocephala* and *N. uenoi*, which exhibit marked intraspecific variation in endophallus shape, are distributed on the Pacific side, whereas *N. niohozana*, *N. furcata*, and *N. chugokuensis*, which do not show such intraspecific variation, are distributed on the Sea of Japan side. This distribution pattern suggests that species on the Pacific and Sea of Japan sides underwent different differentiation processes. For example, species on the Pacific side may have a more ancient origin, and thus more distinct intraspecific variation may have occurred. This hypothesis should be tested in future studies using molecular data.

The gonopore protrusion of species from Shikoku and Kyushu exhibited significant morphological modifications. This finding was unexpected because, although not explicitly stated, this endophallus structure has been considered to show little interspecific morphological variation and to be an unimportant structure in the comparative genital morphology of *N. reflexa* and its related species. Species of some other *Nebria* subgenera are also known to have a gonopore protrusion (Ledoux and Roux 2005), but in all these species, its shape is relatively simple (generally cylindrical or barrel-shaped). Consequently, the morphological modifications of the *N. elephanta* and *N. hikosana* gonopore protrusions are considered to be derived character states. This unexpected finding in *N. elephanta* and *N. hikosana* led to the assumption that interspecific variation in the gonopore protrusion may have been overlooked in previous studies of *N. reflexa* and its allies. Here, to address this issue, the morphology of the gonopore protrusion was re-evaluated based on the photographs shown in previous studies. It became apparent that interspecific variation may show a geographical pattern. For example, in *N. reflexa* and *N. kasasugaensis* Sasakawa & Itô, 2021, which are distributed only in northern Honshu, the endophallus-base side of the gonopore protrusion is distinctly swollen (Sasakawa 2020; Sasakawa and Itô 2021). In three species distributed on the Pacific side of central Honshu—*N. uenoi*, *N. dichotoma*, and *N. dracocephala*—the gonopore protrusion is strongly bent at the base toward the base of the endophallus (Sasakawa 2020, 2023c; this study). In the remaining species, all of which are distributed on the Sea of Japan side, the gonopore protrusion is generally straight and cylindrical (Sasakawa 2020, 2023b). Therefore, in Honshu, species with the same type of gonopore protrusion are geographically clustered, implying that the morphology of the gonopore protrusion may contain phylogenetic information.

On the other hand, the marked morphological modification of the gonopore protrusion in the Shikoku and Kyushu species probably does not provide any information on the phylogenetic relationship between the two species. This is because the gonopore protrusions of the two species are completely different in terms of the region and direction of the modification. It is not yet clear whether *N. elephanta* from Shikoku and *N. hikosana* from Kyushu are sister species. Even if these two species are sister taxa, the developed gonopore protrusion likely evolved independently in each species after their divergence. The morphological diversification of the gonopore protrusion should be investigated in future studies from not only a phylogenetic and taxonomic perspective but also from the perspective of functional morphology. This is because, in some other groups of Carabidae, diversified endophallus structures have been shown to be associated with other reproductive traits, such as mating behavior and ejaculate morphology (e.g., Sasakawa 2006, 2022; Takami and Sota 2007). The marked morphological modification of the *N. elephanta* and *N. hikosana* gonopore protrusions may also be explained as a result of their functional morphology.

## Supplementary Material

XML Treatment for Nebria (Falcinebria) niohozana

XML Treatment for Nebria (Falcinebria) furcata

XML Treatment for Nebria (Falcinebria) dichotoma

XML Treatment for Nebria (Falcinebria) dracocephala

XML Treatment for Nebria (Falcinebria) elephanta

XML Treatment for Nebria (Falcinebria) hikosana

## ﻿Additional information

### Use of AI

No use of AI was reported.

### Author ORCIDs

Kôji Sasakawa https://orcid.org/0000-0001-9246-5777

### Data availability

All of the data that support the findings of this study are available in the main text or Supplementary Information.

## Supplementary materials

Supplementary material 1 Supplementary information Data type docx Explanation note **table S1.** Proposed Japanese names for *N. dracocephala*, *N. elephanta*, and *N. hikosana*. **table S2.** List of the specimens examined. https://binary.pensoft.net/file/1428084This dataset is made available under the Open Database License (http://opendatacommons.org/licenses/odbl/1.0/). The Open Database License (ODbL) is a license agreement intended to allow users to freely share, modify, and use this Dataset while maintaining this same freedom for others, provided that the original source and author(s) are credited.Author: Kôji Sasakawa
