# Supplementary material for: ﻿Taxonomic studies of the ground beetle subgenus Falcinebria Ledoux & Roux, 2005 (Coleoptera, Carabidae, Nebria) from the Japanese Alps (central Honshu), Shikoku, and Kyushu, Japan
Source: Zookeys. 2025 Oct 1;1254:131–48. doi: 10.3897/zookeys.1254.157095 (PMC12508760; doi:10.3897/zookeys.1254.157095)
Supplement: Supplementary material 1 — Supplementary information [file zookeys-1254-131_article-157095__-s001.docx]

Table S1. Proposed Japanese names for *N. dracocephala*, *N. elephanta*, and *N. hikosana*.

| Species | Japanese name |
| --- | --- |
| *N. dracocephala* | Ryûtô-hime-marukubi-gomimushi |
| *N. elephanta* | Tsurugi-hime-marukubi-gomimushi |
| *N. hikosana* | Hikosan-hime-marukubi-gomimushi |

**Table S2.** List of the specimens examined.

| ID | Species | Sex | Locality | Collection_date | Collector | Body length (mm) |
| --- | --- | --- | --- | --- | --- | --- |
| cMMR_0004_M | hikosana | Male | Japan, Saga Prefecture, Kanzaki-shi, Mount Seburi-san, alt. 950 m | 15-V-2001 | Masato Mori | 9.915 |
| cMMR_0005_M | elephanta | Male | Japan, Tokushima Prefecture, Tsurugi-cho, Ichiu, near Tsurugisan Ski Area, alt. 1000 m | 14-VII-2006 | Masato Mori | 9.812 |
| cMMR_0006_M | hikosana | Male | Japan, Saga Prefecture, Kanzaki-shi, Mount Seburi-san, alt. 950 m | 15-V-2001 | Masato Mori | 9.615 |
| cMMR_0007_M | hikosana | Male | Japan, Saga Prefecture, Kanzaki-shi, Mount Seburi-san, alt. 950 m | 15-V-2001 | Masato Mori | 9.833 |
| cMMR_0008_M | hikosana | Male | Japan, Saga Prefecture, Kanzaki-shi, Mount Seburi-san, alt. 950 m | 15-V-2001 | Masato Mori | 9.697 |
| cMMR_0009_F | hikosana | Female | Japan, Saga Prefecture, Kanzaki-shi, Mount Seburi-san, alt. 950 m | 15-V-2001 | Masato Mori | 10.618 |
| cMMR_0010_F | hikosana | Female | Japan, Saga Prefecture, Kanzaki-shi, Mount Seburi-san, alt. 950 m | 15-V-2001 | Masato Mori | 10.265 |
| cMMR_0011_F | hikosana | Female | Japan, Saga Prefecture, Kanzaki-shi, Mount Seburi-san, alt. 950 m | 15-V-2001 | Masato Mori | 10.89 |
| cMMR_0012_F | hikosana | Female | Japan, Saga Prefecture, Kanzaki-shi, Mount Seburi-san, alt. 950 m | 15-V-2001 | Masato Mori | 10.285 |
| cMMR_0013_F | hikosana | Female | Japan, Saga Prefecture, Kanzaki-shi, Mount Seburi-san, alt. 950 m | 15-V-2001 | Masato Mori | 10.716 |
| cMMR_0014_M | elephanta | Male | Japan, Tokushima Prefecture, Higashimiyoshi-cho, Nishisho, near Sajiki-tôge Pass, alt. 800 m | 10-VII-1999 | Masato Mori | 9.697 |
| cMMR_0015_F | elephanta | Female | Japan, Tokushima Prefecture, Higashimiyoshi-cho, Nishisho, near Sajiki-tôge Pass, alt. 800 m | 10-VII-1999 | Masato Mori | 10.542 |
| cMMR_0016_M | dracocephala | Male | Japan, Shizuoka Prefecture, Hamamatsu-shi, Mount Ryûtô-san, alt. 1200 m | 3-X-2006 | [no collector data] | 10.256 |
| cMMR_0017_M | dracocephala | Male | Japan, Shizuoka Prefecture, Shizuoka-shi, Aoi-ku, Mount Sasa-yama, alt. 1500 m | 29-X-2005 | Masato Mori | 9.948 |
| cMMR_0018_F | dracocephala | Female | Japan, Shizuoka Prefecture, Hamamatsu-shi, Mount Ryûtô-san, alt. 1200 m | 3-X-2006 | [no collector data] | 10.893 |
| cMMR_0019_M | furcata | Male | Japan, Gifu Prefecture, Gero-shi, Hagiwaracho, Yamanokuchi, Kuraiyama-tôge Pass, alt. 1100 m | 2-VII-2011 | Kazue Ito | 9.557 |
| cMMR_0020_F | furcata | Female | Japan, Gifu Prefecture, Gero-shi, Hagiwaracho, Yamanokuchi, Kuraiyama-tôge Pass, alt. 1100 m | 2-VII-2011 | Kazue Ito | 10.092 |
| cMMR_0024_M | dichotoma | Male | Japan, Nagano Prefecture, Hiraya-mura, near Hiraya-tôge Pass | 14-VI-2002 | Hiroshi & Takeyuki Nishida | 9.954 |
| cMMR_0025_M | dichotoma | Male | Japan, Nagano Prefecture, Hiraya-mura, near Hiraya-tôge Pass | 14-VI-2002 | Hiroshi & Takeyuki Nishida | 10.384 |
| cMMR_0026_M | dracocephala | Male | Japan, Shizuoka Prefecture, Tenryu-shi, Sakumacho, Mount Idoguchi-yama, the upper reaches of Aizuki-gawa River | 18-V-1996 | Hiroshi Nishida | 10.728 |
| cMMR_0027_M | dracocephala | Male | Japan, Shizuoka Prefecture, Tenryu-shi, Sakumacho, Mount Idoguchi-yama, the upper reaches of Aizuki-gawa River | 18-V-1996 | Hiroshi Nishida | 10.114 |
| cMMR_0028_F | dracocephala | Female | Japan, Shizuoka Prefecture, Tenryu-shi, Sakumacho, Mount Idoguchi-yama, the upper reaches of Aizuki-gawa River | 18-V-1996 | Hiroshi Nishida | 11.128 |
| cMMR_0029_M | niohozana | Male | Japan, Gifu Prefecture, Hida-shi, Kamioka-cho, Utsubo~Toyama Prefecture, Toyama-shi, Arimine, Mount Teraji-yama, Tobikoshi-shindô | 4-X-2008 | Hiroshi Nishida | 10.169 |
| cMMR_0030_M | niohozana | Male | Japan, Gifu Prefecture, Hida-shi, Kamioka-cho, Utsubo~Toyama Prefecture, Toyama-shi, Arimine, Mount Teraji-yama, Tobikoshi-shindô | 4-X-2008 | Hiroshi Nishida | 10.239 |
| cMMR_0031_M | niohozana | Male | Japan, Gifu Prefecture, Hida-shi, Kamioka-cho, Utsubo~Toyama Prefecture, Toyama-shi, Arimine, Mount Teraji-yama, Tobikoshi-shindô | 4-X-2008 | Hiroshi Nishida | 10.135 |
| cMMR_0032_F | niohozana | Female | Japan, Gifu Prefecture, Hida-shi, Kamioka-cho, Utsubo~Toyama Prefecture, Toyama-shi, Arimine, Mount Teraji-yama, Tobikoshi-shindô | 4-X-2008 | Hiroshi Nishida | 10.793 |
| cMMR_0033_F | niohozana | Female | Japan, Gifu Prefecture, Hida-shi, Kamioka-cho, Utsubo~Toyama Prefecture, Toyama-shi, Arimine, Mount Teraji-yama, Tobikoshi-shindô | 4-X-2008 | Hiroshi Nishida | 11.018 |
| cMMR_0034_M | dichotoma | Male | Japan, Aichi Prefecture, Toyota-shi, Otagicho, Dando-dani Valley | 27-V-1989 | Hiroshi & Masami Nishida | 10.158 |
| cMMR_0035_F | dichotoma | Female | Japan, Aichi Prefecture, Toyota-shi, Otagicho, Dando-dani Valley | 27-V-1989 | Hiroshi & Masami Nishida | 10.592 |
| cMMR_0036_F | furcata | Female | Japan, Gifu Prefecture, Gero-shi, Hagiwaracho, Yamanokuchi, Mount Kurai-yama, near the upper reaches of Araragi-ko Lake (Kuguno Disaster Prevention Dam), alt. 1100 m | 18-VI-2009 | Hiroshi Nishida | 9.959 |
| cMMR_0037_F | furcata | Female | Japan, Gifu Prefecture, Gero-shi, Hagiwaracho, Yamanokuchi, Mount Kurai-yama, near the upper reaches of Araragi-ko Lake (Kuguno Disaster Prevention Dam), alt. 1100 m | 18-VI-2009 | Hiroshi Nishida | 10.325 |
| cMMR_0038_F | furcata | Female | Japan, Gifu Prefecture, Gero-shi, Hagiwaracho, Yamanokuchi, Mount Kurai-yama, near the upper reaches of Araragi-ko Lake (Kuguno Disaster Prevention Dam), alt. 1100 m | 18-VI-2009 | Hiroshi Nishida | 9.763 |
| cMMR_0039_F | furcata | Female | Japan, Gifu Prefecture, Gero-shi, Hagiwaracho, Yamanokuchi, Mount Kurai-yama, near the upper reaches of Araragi-ko Lake (Kuguno Disaster Prevention Dam), alt. 1100 m | 18-VI-2009 | Hiroshi Nishida | 9.841 |
| cMMR_0040_F | furcata | Female | Japan, Gifu Prefecture, Gero-shi, Hagiwaracho, Yamanokuchi, Mount Kurai-yama, near the upper reaches of Araragi-ko Lake (Kuguno Disaster Prevention Dam), alt. 1100 m | 18-VI-2009 | Hiroshi Nishida | 9.926 |
| KMNH_KS_013_M | hikosana | Male | Japan, Fukuoka Prefecture, Soeda-machi, Hikosan, Mount Hiko-san, Ryûmon-kyô Gorge | 8-V-1989 | Yasuo Takakura | 9.634 |
| KMNH_KS_014_M | hikosana | Male | Japan, Fukuoka Prefecture, Soeda-machi, Hikosan, Mount Hiko-san | 8-VI-1982 | Yasuo Takakura | 9.141 |
| KMNH_KS_015_M | hikosana | Male | Japan, Fukuoka Prefecture, Soeda-machi, Hikosan, Mount Hiko-san, Ryûmon-kyô Gorge | 8-V-1989 | Yasuo Takakura | 9.904 |
| KMNH_KS_016_F | hikosana | Female | Japan, Fukuoka Prefecture, Fukuoka-shi~Saga Prefecture, Kanzaki-shi, Mount Seburi-san | 7-V-1977 | Munemichi Fukamachi | 10.562 |
| Nebria hikosana Holotype male (COL-005 in NARO) | hikosana | Male | "Col.A.HABU / Mt. Hiko / Fukuoka P. / V. 23, 1950 // Holotype / Nebria / hikosana / HABU // ♂" |  |  | 9.382 |
